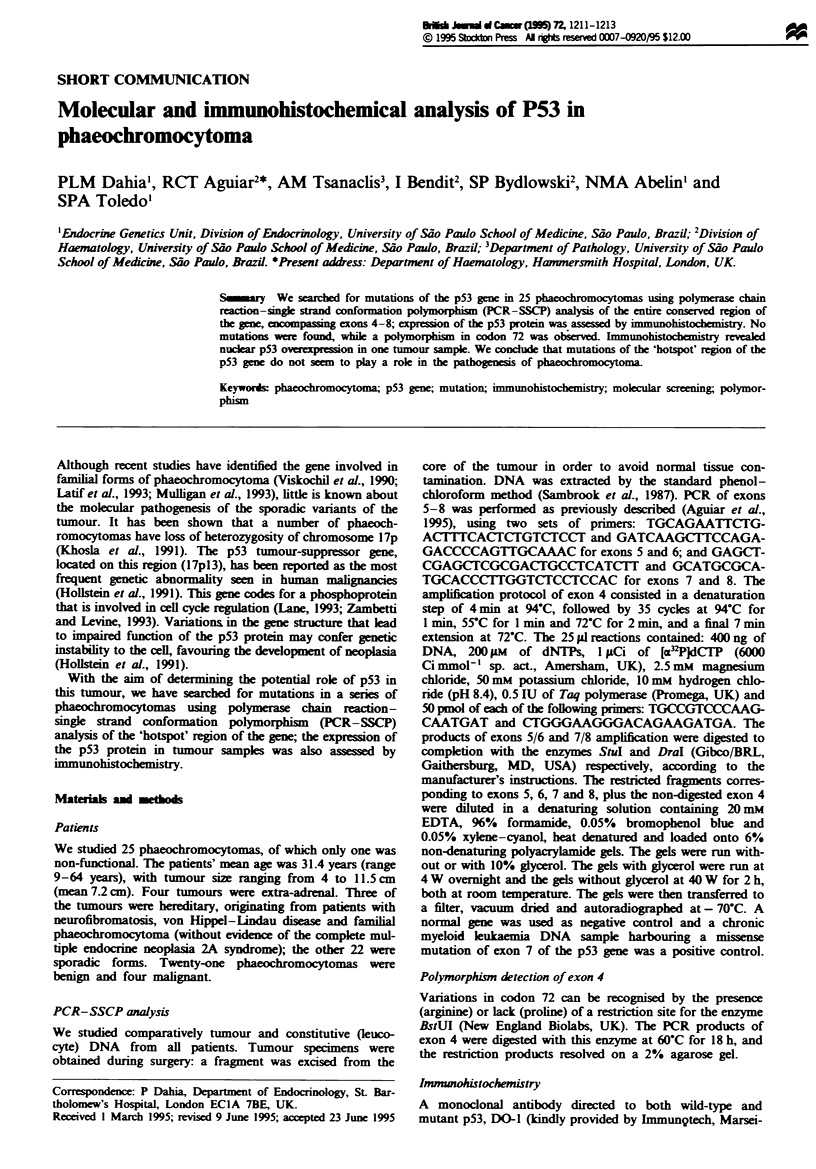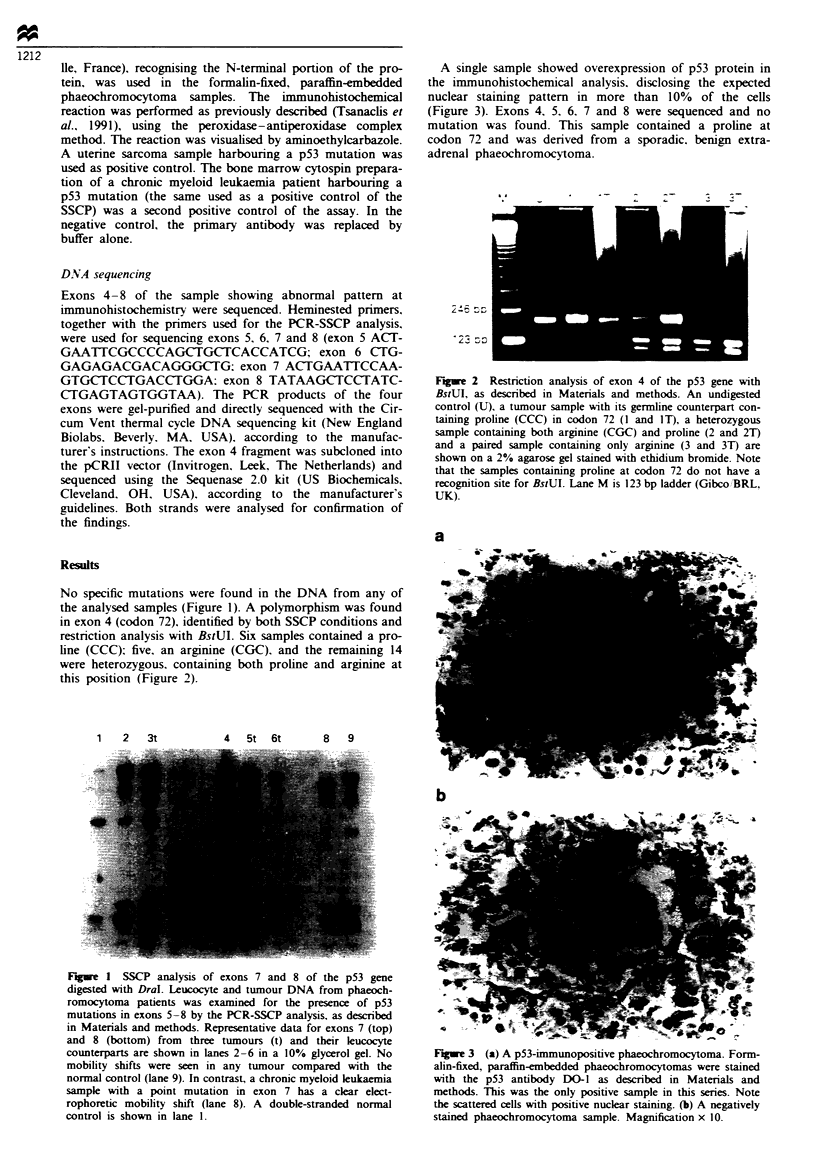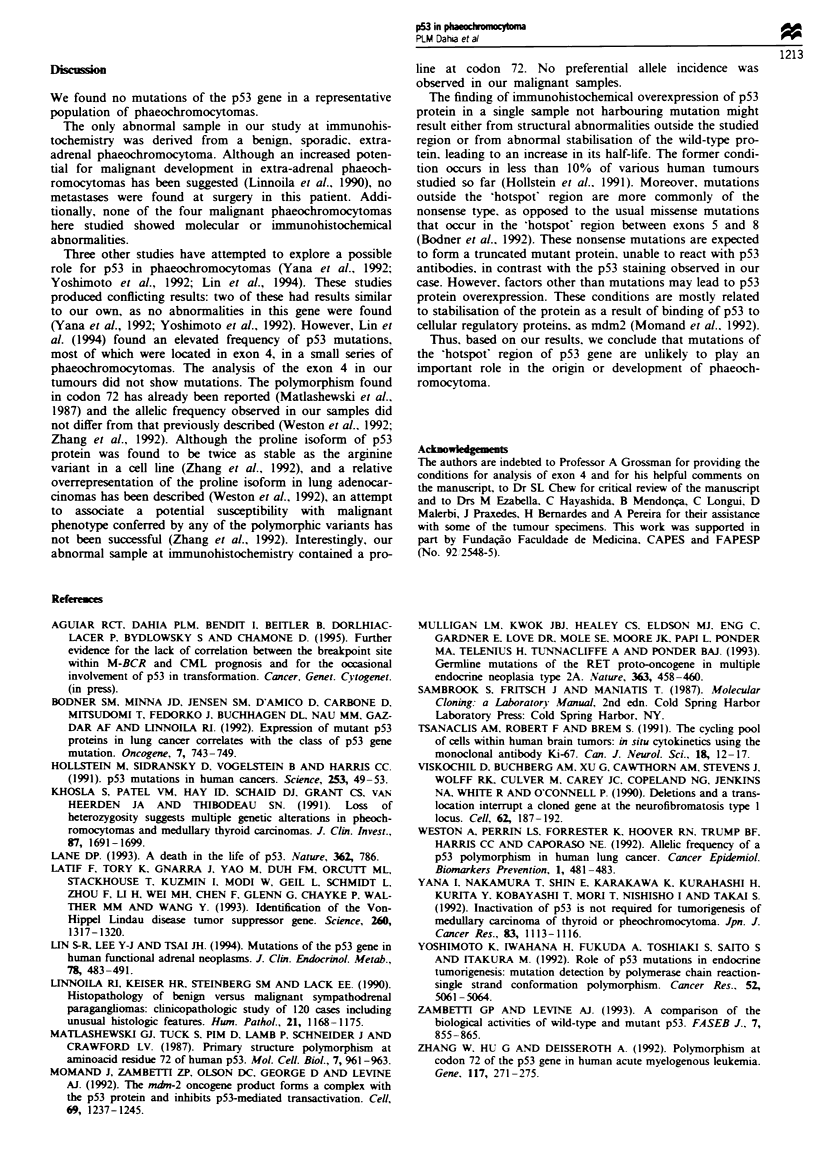# Molecular and immunohistochemical analysis of P53 in phaeochromocytoma.

**DOI:** 10.1038/bjc.1995.487

**Published:** 1995-11

**Authors:** P. L. Dahia, R. C. Aguiar, A. M. Tsanaclis, I. Bendit, S. P. Bydlowski, N. M. Abelin, S. P. Toledo

**Affiliations:** Endocrine Genetics Unit, University of São Paulo School of Medicine, Brazil.

## Abstract

**Images:**


© 1995 Stockton Press All rights reserved 0007-0920/95 $12.00

SHORT COMMUNICATION

### Molecular and immunohistochemical analysis of P53 in phaeochromocytoma

PLM Dahia[1], RCT Aguiar[2*], AM Tsanaclis[3], I Bendit[2], SP Bydlowski[2], NMA Abelin[1] and SPA Toledo[1]

[1]*Endocrine Genetics Unit, Division of Endocrinology, University of São Paulo School of Medicine, São Paulo, Brazil;* [2]*Division of Haematology, University of São Paulo School of Medicine, São Paulo, Brazil;* [3]*Department of Pathology, University of São Paulo School of Medicine, São Paulo, Brazil. *Present address: Department of Haematology, Hammersmith Hospital, London, UK.*

**Summary** We searched for mutations of the p53 gene in 25 phaeochromocytomas using polymerase chain reaction-single strand conformation polymorphism (PCR-SSCP) analysis of the entire conserved region of the gene, encompassing exons 4-8; expression of the p53 protein was assessed by immunohistochemistry. No mutations were found, while a polymorphism in codon 72 was observed. Immunohistochemistry revealed nuclear p53 overexpression in one tumour sample. We conclude that mutations of the 'hotspot' region of the p53 gene do not seem to play a role in the pathogenesis of phaeochromocytoma.

**Keywords:** phaeochromocytoma; p53 gene; mutation; immunohistochemistry; molecular screening; polymorphism

Although recent studies have identified the gene involved in familial forms of phaeochromocytoma (Viskochil *et al.*, 1990; Latif *et al.*, 1993; Mulligan *et al.*, 1993), little is known about the molecular pathogenesis of the sporadic variants of the tumour. It has been shown that a number of phaeochromocytomas have loss of heterozygosity of chromosome 17p (Khosla *et al.*, 1991). The p53 tumour-suppressor gene, located on this region (17p13), has been reported as the most frequent genetic abnormality seen in human malignancies (Hollstein *et al.*, 1991). This gene codes for a phosphoprotein that is involved in cell cycle regulation (Lane, 1993; Zambetti and Levine, 1993). Variations in the gene structure that lead to impaired function of the p53 protein may confer genetic instability to the cell, favouring the development of neoplasia (Hollstein *et al.*, 1991).

With the aim of determining the potential role of p53 in this tumour, we have searched for mutations in a series of phaeochromocytomas using polymerase chain reaction-single strand conformation polymorphism (PCR-SSCP) analysis of the 'hotspot' region of the gene; the expression of the p53 protein in tumour samples was also assessed by immunohistochemistry.

### Materials and methods

### *Patients*

We studied 25 phaeochromocytomas, of which only one was non-functional. The patients' mean age was 31.4 years (range 9-64 years), with tumour size ranging from 4 to 11.5 cm (mean 7.2 cm). Four tumours were extra-adrenal. Three of the tumours were hereditary, originating from patients with neurofibromatosis, von Hippel-Lindau disease and familial phaeochromocytoma (without evidence of the complete multiple endocrine neoplasia 2A syndrome); the other 22 were sporadic forms. Twenty-one phaeochromocytomas were benign and four malignant.

### *PCR-SSCP analysis*

We studied comparatively tumour and constitutive (leucocyte) DNA from all patients. Tumour specimens were obtained during surgery: a fragment was excised from the core of the tumour in order to avoid normal tissue contamination. DNA was extracted by the standard phenol-chloroform method (Sambrook *et al.*, 1987). PCR of exons 5-8 was performed as previously described (Aguiar *et al.*, 1995), using two sets of primers: TGCAGAATTCTG-ACTTTCACTCTGTCTCCT and GATCAAGCTTCCAGA-GACCCCAGTTGCAAAC for exons 5 and 6; and GAGCT-CGAGCTCGCGACTGCCTCATCTT and GCATGCGCA-TGCACCCTTGGTCTCCTCCAC for exons 7 and 8. The amplification protocol of exon 4 consisted in a denaturation step of 4 min at 94°C, followed by 35 cycles at 94°C for 1 min, 55°C for 1 min and 72°C for 2 min, and a final 7 min extension at 72°C. The 25 µl reactions contained: 400 ng of DNA, 200 µM of dNTPs, 1 µCi of [$\alpha$-$^{32}$P]dCTP (6000 Ci mmol$^{-1}$ sp. act., Amersham, UK), 2.5 mM magnesium chloride, 50 mM potassium chloride, 10 mM hydrogen chloride (pH 8.4), 0.5 IU of *Taq* polymerase (Promega, UK) and 50 pmol of each of the following primers: TGCCGTCCCAAG-CAATGAT and CTGGGAAGGGACAGAAGATGA. The products of exons 5/6 and 7/8 amplification were digested to completion with the enzymes *Stu*I and *Dra*I (Gibco/BRL, Gaithersburg, MD, USA) respectively, according to the manufacturer's instructions. The restricted fragments corresponding to exons 5, 6, 7 and 8, plus the non-digested exon 4 were diluted in a denaturing solution containing 20 mM EDTA, 96% formamide, 0.05% bromophenol blue and 0.05% xylene-cyanol, heat denatured and loaded onto 6% non-denaturing polyacrylamide gels. The gels were run without or with 10% glycerol. The gels with glycerol were run at 4 W overnight and the gels without glycerol at 40 W for 2 h, both at room temperature. The gels were then transferred to a filter, vacuum dried and autoradiographed at − 70°C. A normal gene was used as negative control and a chronic myeloid leukaemia DNA sample harbouring a missense mutation of exon 7 of the p53 gene was a positive control.

### *Polymorphism detection of exon 4*

Variations in codon 72 can be recognised by the presence (arginine) or lack (proline) of a restriction site for the enzyme *Bst*UI (New England Biolabs, UK). The PCR products of exon 4 were digested with this enzyme at 60°C for 18 h, and the restriction products resolved on a 2% agarose gel.

### *Immunohistochemistry*

A monoclonal antibody directed to both wild-type and mutant p53, DO-1 (kindly provided by Immunotech, Marsei-

Correspondence: P Dahia, Department of Endocrinology, St. Bartholomew's Hospital, London EC1A 7BE, UK.
Received 1 March 1995; revised 9 June 1995; accepted 23 June 1995

lle, France), recognising the N-terminal portion of the protein, was used in the formalin-fixed, paraffin-embedded phaeochromocytoma samples. The immunohistochemical reaction was performed as previously described (Tsanaclis *et al*., 1991), using the peroxidase-antiperoxidase complex method. The reaction was visualised by aminoethylcarbazole. A uterine sarcoma sample harbouring a p53 mutation was used as positive control. The bone marrow cytospin preparation of a chronic myeloid leukaemia patient harbouring a p53 mutation (the same used as a positive control of the SSCP) was a second positive control of the assay. In the negative control, the primary antibody was replaced by buffer alone.

### *DNA sequencing*

Exons 4-8 of the sample showing abnormal pattern at immunohistochemistry were sequenced. Heminested primers, together with the primers used for the PCR-SSCP analysis, were used for sequencing exons 5, 6, 7 and 8 (exon 5 ACTGAATTCGCCCCAGCTGCTCACCATCG; exon 6 CTGGAGAGACGACAGGGCTG; exon 7 ACTGAATTCCAAGTGCTCCTGACCTGGA; exon 8 TATAAGCTCCTATCCTGAGTAGTGGTAA). The PCR products of the four exons were gel-purified and directly sequenced with the Circum Vent thermal cycle DNA sequencing kit (New England Biolabs, Beverly, MA, USA), according to the manufacturer's instructions. The exon 4 fragment was subcloned into the pCRII vector (Invitrogen, Leek, The Netherlands) and sequenced using the Sequenase 2.0 kit (US Biochemicals, Cleveland, OH, USA), according to the manufacturer's guidelines. Both strands were analysed for confirmation of the findings.

### Results

No specific mutations were found in the DNA from any of the analysed samples (Figure 1). A polymorphism was found in exon 4 (codon 72), identified by both SSCP conditions and restriction analysis with *Bst*UI. Six samples contained a proline (CCC); five, an arginine (CGC), and the remaining 14 were heterozygous, containing both proline and arginine at this position (Figure 2).

**Figure 1** SSCP analysis of exons 7 and 8 of the p53 gene digested with *DraI*. Leucocyte and tumour DNA from phaeochromocytoma patients was examined for the presence of p53 mutations in exons 5-8 by the PCR-SSCP analysis, as described in Materials and methods. Representative data for exons 7 (top) and 8 (bottom) from three tumours (t) and their leucocyte counterparts are shown in lanes 2-6 in a 10% glycerol gel. No mobility shifts were seen in any tumour compared with the normal control (lane 9). In contrast, a chronic myeloid leukaemia sample with a point mutation in exon 7 has a clear electrophoretic mobility shift (lane 8). A double-stranded normal control is shown in lane 1.

A single sample showed overexpression of p53 protein in the immunohistochemical analysis, disclosing the expected nuclear staining pattern in more than 10% of the cells (Figure 3). Exons 4, 5, 6, 7 and 8 were sequenced and no mutation was found. This sample contained a proline at codon 72 and was derived from a sporadic, benign extra-adrenal phaeochromocytoma.

**Figure 2** Restriction analysis of exon 4 of the p53 gene with *Bst*UI, as described in Materials and methods. An undigested control (U), a tumour sample with its germline counterpart containing proline (CCC) in codon 72 (1 and 1T), a heterozygous sample containing both arginine (CGC) and proline (2 and 2T) and a paired sample containing only arginine (3 and 3T) are shown on a 2% agarose gel stained with ethidium bromide. Note that the samples containing proline at codon 72 do not have a recognition site for *Bst*UI. Lane M is 123 bp ladder (Gibco/BRL, UK).

**Figure 3** (**a**) A p53-immunopositive phaeochromocytoma. Formalin-fixed, paraffin-embedded phaeochromocytomas were stained with the p53 antibody DO-1 as described in Materials and methods. This was the only positive sample in this series. Note the scattered cells with positive nuclear staining. (**b**) A negatively stained phaeochromocytoma sample. Magnification × 10.

### Discussion

We found no mutations of the p53 gene in a representative population of phaeochromocytomas.

The only abnormal sample in our study at immunohistochemistry was derived from a benign, sporadic, extra-adrenal phaeochromocytoma. Although an increased potential for malignant development in extra-adrenal phaeochromocytomas has been suggested (Linnoila *et al*., 1990), no metastases were found at surgery in this patient. Additionally, none of the four malignant phaeochromocytomas here studied showed molecular or immunohistochemical abnormalities.

Three other studies have attempted to explore a possible role for p53 in phaeochromocytomas (Yana *et al*., 1992; Yoshimoto *et al*., 1992; Lin *et al*., 1994). These studies produced conflicting results: two of these had results similar to our own, as no abnormalities in this gene were found (Yana *et al*., 1992; Yoshimoto *et al*., 1992). However, Lin *et al*. (1994) found an elevated frequency of p53 mutations, most of which were located in exon 4, in a small series of phaeochromocytomas. The analysis of the exon 4 in our tumours did not show mutations. The polymorphism found in codon 72 has already been reported (Matlashewski *et al*., 1987) and the allelic frequency observed in our samples did not differ from that previously described (Weston *et al*., 1992; Zhang *et al*., 1992). Although the proline isoform of p53 protein was found to be twice as stable as the arginine variant in a cell line (Zhang *et al*., 1992), and a relative overrepresentation of the proline isoform in lung adenocarcinomas has been described (Weston *et al*., 1992), an attempt to associate a potential susceptibility with malignant phenotype conferred by any of the polymorphic variants has not been successful (Zhang *et al*., 1992). Interestingly, our abnormal sample at immunohistochemistry contained a proline at codon 72. No preferential allele incidence was observed in our malignant samples.

The finding of immunohistochemical overexpression of p53 protein in a single sample not harbouring mutation might result either from structural abnormalities outside the studied region or from abnormal stabilisation of the wild-type protein, leading to an increase in its half-life. The former condition occurs in less than 10% of various human tumours studied so far (Hollstein *et al*., 1991). Moreover, mutations outside the 'hotspot' region are more commonly of the nonsense type, as opposed to the usual missense mutations that occur in the 'hotspot' region between exons 5 and 8 (Bodner *et al*., 1992). These nonsense mutations are expected to form a truncated mutant protein, unable to react with p53 antibodies, in contrast with the p53 staining observed in our case. However, factors other than mutations may lead to p53 protein overexpression. These conditions are mostly related to stabilisation of the protein as a result of binding of p53 to cellular regulatory proteins, as mdm2 (Momand *et al*., 1992).

Thus, based on our results, we conclude that mutations of the 'hotspot' region of p53 gene are unlikely to play an important role in the origin or development of phaeochromocytoma.

**Acknowledgements**
The authors are indebted to Professor A Grossman for providing the conditions for analysis of exon 4 and for his helpful comments on the manuscript, to Dr SL Chew for critical review of the manuscript and to Drs M Ezabella, C Hayashida, B Mendonça, C Longui, D Malerbi, J Praxedes, H Bernardes and A Pereira for their assistance with some of the tumour specimens. This work was supported in part by Fundação Faculdade de Medicina, CAPES and FAPESP (No. 92/2548-5).

### References

AGUIAR RCT, DAHIA PLM, BENDIT I, BEITLER B, DORLHIAC-LACER P, BYDLOWSKY S AND CHAMONE D. (1995). Further evidence for the lack of correlation between the breakpoint site within M-*BCR* and CML prognosis and for the occasional involvement of p53 in transformation. *Cancer, Genet. Cytogenet*. (in press).

BODNER SM, MINNA JD, JENSEN SM, D'AMICO D, CARBONE D, MITSUDOMI T, FEDORKO J, BUCHHAGEN DL, NAU MM, GAZDAR AF AND LINNOILA RI. (1992). Expression of mutant p53 proteins in lung cancer correlates with the class of p53 gene mutation. *Oncogene*, **7**, 743–749.

HOLLSTEIN M, SIDRANSKY D, VOGELSTEIN B AND HARRIS CC. (1991). p53 mutations in human cancers. *Science*, **253**, 49–53.

KHOSLA S, PATEL VM, HAY ID, SCHAID DJ, GRANT CS, VAN HEERDEN JA AND THIBODEAU SN. (1991). Loss of heterozygosity suggests multiple genetic alterations in pheochromocytomas and medullary thyroid carcinomas. *J. Clin. Invest*., **87**, 1691–1699.

LANE DP. (1993). A death in the life of p53. *Nature*, **362**, 786.

LATIF F, TORY K, GNARRA J, YAO M, DUH FM, ORCUTT ML, STACKHOUSE T, KUZMIN I, MODI W, GEIL L, SCHMIDT L, ZHOU F, LI H, WEI MH, CHEN F, GLENN G, CHAYKE P, WALTHER MM AND WANG Y. (1993). Identification of the Von-Hippel Lindau disease tumor suppressor gene. *Science*, **260**, 1317–1320.

LIN S-R, LEE Y-J AND TSAI JH. (1994). Mutations of the p53 gene in human functional adrenal neoplasms. *J. Clin. Endocrinol. Metab*., **78**, 483–491.

LINNOILA RI, KEISER HR, STEINBERG SM AND LACK EE. (1990). Histopathology of benign versus malignant sympathodrenal paragangliomas: clinicopathologic study of 120 cases including unusual histologic features. *Hum. Pathol*., **21**, 1168–1175.

MATLASHEWSKI GJ, TUCK S, PIM D, LAMB P, SCHNEIDER J AND CRAWFORD LV. (1987). Primary structure polymorphism at aminoacid residue 72 of human p53. *Mol. Cell. Biol*., **7**, 961–963.

MOMAND J, ZAMBETTI ZP, OLSON DC, GEORGE D AND LEVINE AJ. (1992). The *mdm*-2 oncogene product forms a complex with the p53 protein and inhibits p53-mediated transactivation. *Cell*, **69**, 1237–1245.

MULLIGAN LM, KWOK JBJ, HEALEY CS, ELDSON MJ, ENG C, GARDNER E, LOVE DR, MOLE SE, MOORE JK, PAPI L, PONDER MA, TELENIUS H, TUNNACLIFFE A AND PONDER BAJ. (1993). Germline mutations of the RET proto-oncogene in multiple endocrine neoplasia type 2A. *Nature*, **363**, 458–460.

SAMBROOK S, FRITSCH J AND MANIATIS T. (1987). *Molecular Cloning: a Laboratory Manual*, 2nd edn. Cold Spring Harbor Laboratory Press: Cold Spring Harbor, NY.

TSANACLIS AM, ROBERT F AND BREM S. (1991). The cycling pool of cells within human brain tumors: *in situ* cytokinetics using the monoclonal antibody Ki-67. *Can. J. Neurol. Sci*., **18**, 12–17.

VISKOCHIL D, BUCHBERG AM, XU G, CAWTHORN AM, STEVENS J, WOLFF RK, CULVER M, CAREY JC, COPELAND NG, JENKINS NA, WHITE R AND O'CONNELL P. (1990). Deletions and a translocation interrupt a cloned gene at the neurofibromatosis type 1 locus. *Cell*, **62**, 187–192.

WESTON A, PERRIN LS, FORRESTER K, HOOVER RN, TRUMP BF, HARRIS CC AND CAPORASO NE. (1992). Allelic frequency of a p53 polymorphism in human lung cancer. *Cancer Epidemiol. Biomarkers Prevention*, **1**, 481–483.

YANA I, NAKAMURA T, SHIN E, KARAKAWA K, KURAHASHI H, KURITA Y, KOBAYASHI T, MORI T, NISHISHO I AND TAKAI S. (1992). Inactivation of p53 is not required for tumorigenesis of medullary carcinoma of thyroid or pheochromocytoma. *Jpn. J. Cancer Res*., **83**, 1113–1116.

YOSHIMOTO K, IWAHANA H, FUKUDA A, TOSHIAKI S, SAITO S AND ITAKURA M. (1992). Role of p53 mutations in endocrine tumorigenesis: mutation detection by polymerase chain reaction-single strand conformation polymorphism. *Cancer Res*., **52**, 5061–5064.

ZAMBETTI GP AND LEVINE AJ. (1993). A comparison of the biological activities of wild-type and mutant p53. *FASEB J*., **7**, 855–865.

ZHANG W, HU G AND DEISSEROTH A. (1992). Polymorphism at codon 72 of the p53 gene in human acute myelogenous leukemia. *Gene*, **117**, 271–275.